# The Long Fox Lecture: The Conditioned Response

**Published:** 1924-10

**Authors:** C. Lloyd Morgan

**Affiliations:** Emeritus Professor of Psychology, Ethics and Logic, University of Bristol


The Bristol

Medico-Chirurgical Journal

" Scire est nescire, nisi id me
Scire alius sciret."

OCTOBER, 1924.

### THE LONG FOX LECTURE:

DELIVERED IN THE UNIVERSITY OF BRISTOL ON JULY 23RD, 1924.
PROFESSOR F. FRANCIS, D.Sc., *Pro-Vice-Chancellor in the Chair.*

BY

C. LLOYD MORGAN, LL.D., D.Sc., F.R.S.,
*Emeritus Professor of Psychology, Ethics and Logic, University of Bristol,*

ON

### THE CONDITIONED RESPONSE.

---

In some interesting experiments conducted by Dr. E. J. Allen at the Plymouth Marine Biological Laboratory electric bells were suspended in the tanks in which such fishes as pollack and rudd are kept. At first there was no evidence that the fish were affected by the vibrations transmitted through the water. Food was then placed day after day in the neighbourhood of the ringing bell. After a time—a month or so—when the bell was set in vibration, with no food near, the fish assembled round it apparently in search of the customary food. Two bells, some twelve feet apart, were fitted in the tank. By ringing them alternately the fish could be drawn to and fro in the tank towards that bell which was in vibration.

It seems, then, that the fish were stimulated by the vibrations in the water in some such way as we should speak of as hearing the bell. It seems that what we speak of as an "association" was established between the sound of the bell and the sight and taste of food. It seems that there was expectation of food when the bell was rung, and that the behaviour of the fish was guided by such expectation.

This is a conditioned response—learnt in the course of individual life. The fish, as we say, profited by "the lessons of experience." We naturally interpret this in mental terms—hearing the sound of the bell, sight and taste of food, "association of ideas," expectation, and so on. But we know very little about the mind of a fish. And in any case, such mental processes as may be present can only be surmised from that which is observed. What was observed in Dr. Allen's fish is a new manner of response in behaviour under certain conditions of external stimulation; and what may be inferred is some physiological provision for the establishment of a conditioned response, one acquired in the course of individual life.

Such new ways of response in behaviour have for long been matters of familiar observation. Young chicks peck at any small thing within pecking range with much accuracy. Let us call this a *primary response* to visual stimulation, and let us distinguish the *first occasion* on which such a primary response is in evidence. There may be first occasions in this sense on which the chick pecks at a number of different things, but on each occasion the primary response, the act of pecking, is closely similar. Now let there be, under experimental inquiry, just two different kinds of things, say, maggots and ladybirds. The primary response is to peck at them—either of them without preference—at sight; and so far there is no observable difference in the primary response. The ladybird or maggot is seized. There is thus afforded stimulation in the mouth. The maggot is swallowed; the ladybird is rejected or tossed on one side. Each is a primary response in behaviour and is observable on the first occasion. So far we have different primary responses to different modes of stimulation. But keep the chicks under observation and watch their further behaviour. On sight of another maggot the chick pecks at it, seizes and swallows it with what seems to be added zest; but on sight of another ladybird the primary response to the visual stimulation is checked, and the behaviour of rejection is carried out, though there is no stimulation in the mouth to give this as a primary response. It is a *secondary response* elicited at sight on *subsequent occasions*. Such a secondary response is now termed a "conditioned response."

It may be said that this may be quite simply explained. The chick has profited by experience. He found that the ladybird is nasty, and therefore does not peck at it again. He found that the maggot is nice, and therefore eats as many as he can get. Yes. But how, on physiological grounds, does the niceness and nastiness take effect? What is the organic provision in the structure of the chick for that which observably happens? In what manner is the secondary response conditioned?

From the physiological standpoint the emphasis is on response to stimulation. Hence, as Professor Dendy says: "It must be admitted that the entire life of any organism consists of a series of responses to stimuli which reach it from various sources." Thus all embryological development, all modes of growth, all forms of behaviour, call for interpretation on these terms. But it is probably only when a complex nervous sytem has thus been developed that there is the requisite physiological provision for the conditioned response.

The central nervous system—spinal cord and brain—sends forth nerve-fibres to all parts of the body. Some of these fibres transmit what we may call nerve-events inwards to the central nervous system, others transmit nerve-events outwards from the central nervous system. Thus when the chick sees a ladybird nerve-events are transmitted inwards from the retina. Out there is the ladybird. May I call it a pattern of events in the external world? By it the retina is stimulated. Shall we speak of a *receptor-pattern* of events in the retinal image? There is then a pattern of nerve-events which course inwards along many nerve-fibres to the brain. The chick pecks in primary response. There is a pattern of nerve-events running outwards along many nerve-fibres to the muscles. They call into play what are termed "effectors," since their effect (in this case) is muscular contraction. There is therefore an *effector-pattern* of events, which gives rise to a pattern of muscular change producing that form of behaviour we call pecking. So we have a very complicated incoming set of patterns of events, and a very complicated outgoing set of patterns of events. And connecting them is a central pattern of events in the brain.

Now the neuronic structure of the central nervous system is such that there seems to be a labyrinth of synaptic connections between any one incoming set of events and any other outgoing set of events. But when the chick pecks in primary response the events in the brain seem to follow a quite definite route—from the receptor-pattern in the retina to the effector-pattern in those muscles that are concerned in the behaviour of pecking. It seems, therefore, that this is initially a *more permeable* route than any other route. We have, then, to reckon with a *pattern of*

*permeability* in the brain or central nervous system. A primary response affords evidence of an initially permeable route connecting the incoming with the outgoing sets of events.

When the ladybird is seized there is a gustatory receptor-pattern in the mouth. Nerve-events course inwards. They find in the central nervous system an initially permeable route; and sets of nerve events course outwards to give the primary response of rejection. There seems to be no initial connection between *this* primary route and *that.* It is the establishment of a secondary connection between them that characterises the conditioned response. This secondary connection, affording what is now a more permeable route in the central nervous system, is acquired—sometimes slowly as in Dr. Allen's fish, sometimes very rapidly as in the chick—but always acquired in the course of individual life.

It may still be said that it is mentally and not physiologically acquired. Let us then briefly consider the case of salivation in the dog. It is to Professor Pavlov's work in this field of inquiry that we owe the concept of the conditioned response as he named it. When a dog is fed gustatory stimulation in the mouth induces a flow of saliva. That is part of the primary response. There is also motor response in mastication and so forth, but we here concentrate attention on the response of the salivary glands. There are, however, many other primary responses in the dog; for example, pricking of the ears when there is auditory stimulation. And initially there seems to be no connection between any two of such primary responses. But let the dog receive auditory stimulation from some definite sound concurrently with gustatory stimulation from food in the mouth on a certain number of occasions. Then what happens is that the sound by itself induces a flow of saliva

(which can be measured by inserting a cannula into the salivary duct) *without any gustatory stimulation*. A secondary connection seems to be established in the course of the experiments; and this affords a route of relatively greater permeability, or, in technical terms, of lessened "synaptic resistance." And since salivation is hardly at all (if at all) under conscious control; since, too, the nature of the investigation (into which I cannot here enter) seems to preclude mental influence in the usual sense of this expression; we appear to dig down to a purely physiological interpretation of this conditional response.

Some diagrams (not pictures) may here be helpful. Let *g* be the gustatory "pattern" and *au* the auditory "pattern." And let G be the initially permeable route from *g* in the central nervous system; Au the initially permeable route from *au*. Then we have on the earlier occasions, as synchronous—

*g*— G —salivation.
*au*—Au—pricking of ears.

But on the subsequent or later occasions we have—

G —salivation.
|
*au*—Au

There is no gustatory stimulation, and the pricking of the ears is (as I understand) inhibited. Nerve-events run their course from *au*, through Au to G, and thus to the salivary glands.

We may now give as a general rubric for the conditioned response some such statement as this. Let two primary responses be elicited simultaneously or in close succession on several occasions, then if, sooner or later, the one response is elicited by the *other* kind of stimulation there is a conditioned response, and there is presumptive evidence of the establishment of a secondary route of acquired permeability within the central nervous system.

It may be asked why the route between Au and G is rendered more permeable when these brain-centres are in synchrous activity. Perhaps we do not as yet fully know. But that it does become more permeable seems to be a legitimate inference from a large body of physiological facts. And, did time permit, sundry physical analogies of the increase of the dominant activity in G by some concurrent activity in other parts of an integral system might be adduced.

When the chick rejects a ladybird "at sight" without needing to experience again its presumably nasty taste, we may express the physiological conditions thus:

Sight of l.b.—sight-route—seizing (which entails taste).
Taste of l.b.—taste-route—rejection.

This occurs on the first occasion. But on some subsequent occasion the visual nerve-events are switched off on to the taste-route, and then we have—

Sight of l.b.—sight-route.
|
taste-route—rejection.

In the case of the maggot-situation we have—

Sight of m.—sight-route—seizing (which entails taste).
Taste of m.—taste-route—swallowing.

And on subsequent occasions the taste-response does not thwart but endorses the sight-response. Thus we have—

Sight of m.—sight-route }
Taste of m.—taste-route } —seizing and swallowing.

Under observation we then say that seizing and swallowing occur with "added zest."

Thus we have two types of the conditioned response: (*a*) that in which conditioning thwarts or inhibits some primary response and leads to its *suppression;* (*b*) that in which conditioning supports, endorses or enhances some primary response and leads to its fuller and more vigorous *expression.*

There is no call to deny—nay, rather good ground for asserting—that in many of the conditioned responses of the more highly developed animals there is at least an accom paniment of conscious enjoyment. In seeking physiological foundations one need not be disregarding mental events; one is trying to render clear what nerve processes may be inferred as the correlates of incipient mental processes. And one may find here a noteworthy turning-point in evolutionary advance. To this I shall return.

I want first to bid you remember how highly differentiated is the structure of the organism and of the brain, and how very complicated is the assemblage of physiological events therein. The structure of the neuronic system may be roughly likened to a meshwork of railway lines; the current nerve-events to the trains that are running. As there are thousands of trains that are running at the same time; so too there are thousands of nerve-events, each pursuing some primary course or a conditioned course switched off at certain "points" on the structural lines of rail. We have also to bear in mind: (1) that the sense-organs afford different kinds of external receptor-patterns which may co-exist at the same time; (2) that the outcome of this or that form of behaviour generally gives a new receptor-pattern which starts a new course of nerve-events; (3) that there are not only external receptor-patterns but also internal receptor-patterns registering the activity of the muscles and glands, and the existing state of the living organism; and (4) that all the sets of events are subject to the physiological poise of the time-being, largely determined by the subtly-varying activity of the endocrine glands. If, then, one concentrates attention on some one conditioned response, one should do so with full realisation of the vast number of concurrent physiological events.

I cannot here deal with so widely extended an assemblage of fluent changes. It may, however, be helpful to look just a little more closely into the pecking response which I have selected for illustration.

What one notices under careful observation is that pecking, even if under this head there be included the preparatory posture of the body, is seldom the first effect in responsive behaviour of a visual receptor-pattern—the situation as presented in vision. Straightway pecking only occurs when the object is at just the right distance. At a little greater distance the first obvious response is a step or two forwards till the object is within pecking range. There is thus an enchained sequence of primary responses that lead up to the act of pecking. But what is the first link in the chain? I have devoted a good deal of attention to this question, for I deem it very important. I am convinced that the first link in the chain is the primary response of a clinging of gaze to the small object, and this clinging is maintained during the step or two forward. I believe it to be a physiological response, as are all primary responses. It is seen in the human infant within the first fortnight after birth, and it affords the foundations for the gradual development of focussed vision in the child—well described by Miss Millicent Shinn in *The Biography of a Baby*.

Aversion of gaze from too brightly illuminated objects is likewise a primary response. And unquestionably, in the chick, not-clinging, which is a step towards aversion of gaze, is a determining factor in that apparent ignoring of such things as ladybirds, soldier-beetles, or cinnabar caterpillars, which comes in due course. The clinging of gaze seems to be broken by the shaking of the head, perhaps wiping of the bill on the ground, which the primary response of rejection entails. But since not-clinging, or the fuller aversion of gaze, is not from something which is hurtful to the eye but from something that has been unpleasant to the taste, it is so far a conditioned response acquired in the course of individual life. It is a matter that requires nice and careful observation; but my impression, after prolonged inquiry, is that there may be seen in young birds an initial turning of the gaze away from that the like of which has been unpleasant. And I feel sure that it may be detected in the infant when something nasty comes into the field of vision.

Now clinging of gaze is a primary form of that which will, later on, develop into conscious attention and interest. We seem to be justified in the belief, no doubt inferential, that it is accompanied by pleasurable enjoyment. So, too, aversion of gaze seems to betoken some felt discomfort. Indeed, we commonly speak in a wide sense of "aversion" from that which we have found to be unpleasant.

We thus pass to the psychological bearing of what we have learnt with regard to the conditioned response. Let me here say that I am one of those who believe that all physiological events are accompanied by what I call enjoyment. Thus there are thousands of events all through one's body which are accompanied by that kind of enjoyment which we call "feeling fit" or the reverse. I think that *all* the physiological events that are going on inside us contribute to the enjoyment which affords the foundations on which our minds are built. I use the word "enjoyment," in company with Professor Alexander, to include both pleasurable (positive) and unpleasant (negative) modes of feeling. This seems awkward at first, but so, too, does the use, in physics, of the word "acceleration" for diminution as well as for increase of speed. One soon gets accustomed to the use of a word in a technical sense rather different from that of common parlance. On this understanding all the physiological events that are concerned in primary responses are accompanied by enjoyment positive or negative. But in primary responses it is accompaniment only. What I seek to show is that the conditioned response introduces a new factor of conscious *guidance* towards pleasurable situations and away from situations fraught with discomfort.

Let us here recall the common-sense view that, in my illustrative examples, the chick rejects or avoids ladybirds because they are unpleasantly nasty, and eats all the maggots he can get because they are nice and pleasantly satisfy the cravings of hunger correlated with an internal " pattern " of events. I suppose common sense would speak of this as conscious guidance towards what is pleasant and away from that which is unpleasant; and I think that common sense is so far right. But may not the physiologist who deals with conditioned responses also be right? If so, we have to connect their different points of view.

When we pass from physiological events and processes to conscious events and processes we introduce the notion of *reference*. What does this mean? It means that, if we impute conscious processes to the chick, what the little bird has in its little mind as the result of being stimulated by some physical influence *from* the ladybird, and as the result of acting *on* the ladybird, is referred *to* the ladybird which it sees, pecks at, seizes, and tastes. Thus, from the physical point of view the ladybird is a centre of *influence* on the chick's body; and from the conscious point of view is a centre of *reference* from the chick's mind. We must just accept such reference as a natural fact. It is that which characterises all conscious life as we experience it in ourselves. One is doing no violence to the usage of familiar thought and speech in saying that when one receives light-influence from a thing one also refers what one thus experiences to the object of vision.

But when the chick receives light-influence from the ladybird, and when a set of nerve-events run their course along what I called the sight-route, there is, in the conditioned response, a switching off of the nerve-events on to the taste-route. The ladybird, however, is not then and there tasted. There is no gustatory "pattern" in the mouth. We then speak of the taste-events, called up within the central nervous system, as due to *revival*. There is physiological revival of nerve-events on the taste-route, and there is conscious or felt revival of the enjoyment that accompanies these nerve-events. Three things must here be noted: first, that, judging from our own experience, the mental taste-revival is referred to the ladybird; secondly, that it takes the form of expectation of what may follow; and, thirdly, that this present expectation (for the expectation is *present* though it has prospective *reference*) is either pleasurable or unpleasant.

So far it just comes to this. What the psychologist, on his part, speaks of as expectant revival having reference to the ladybird, with negative enjoyment, the physiologist, on his part, interprets as a conditioned set of nerve-events along part at least of the taste-route.

Now comes the point to which I invite special attention. What we have on "first occasions" is, in order of sequence: (1) sight; (2) pecking; (3) tasting; (4) (*a*) swallowing (maggot), (*b*) rejecting (ladybird).

Here the fourth stage is "this" or "that"—(*a*) or (*b*)—according as there is this or that kind of gustatory "pattern." But on "subsequent occasions," when a conditioned response is established, we have a different *and partly inverted order*, namely: (1) sight; (2) taste-revival; (3) (*a*) pecking, (*b*) not-pecking; (4) (*a*) swallowing, (*b*) rejecting.

The point for emphasis is that here taste-revival is brought forward to the second place in the sequence. Whereas actual tasting *follows* pecking, taste-revival, which we may call "foretaste," *precedes* pecking or not-pecking. This act may then either be endorsed so as to lead on to swallowing, or it may be inhibited, more or less completely, and replaced by the act of rejection. And which of the two acts follows is determined by the character of the foretaste, nice or nasty as the case may be.

It is this inversion of order, entailed by the conditioned response, that introduces a quite new factor, that of conscious guidance, or that which one may call orientation of behaviour towards those situations of which the foretaste is pleasurable, and away from those situations which, in foretaste, are fraught with discomfort. It should, however, be noted that this does not imply determination of present action by the future (for the future is not yet) ; it implies determination of future action by present foretaste due to actual tasting in the past. Now broaden the notion of foretaste so as to include *any* kind of enjoyment, *e.g.* that of the concert for which we have secured tickets ; then the suggestion is that how we act turns on the pleasant and attractive, or the unpleasant and repellent, nature of the foretaste in enjoyment.

I turn in conclusion to a different question. There has been a long controversy as to what is called the inheritance of acquired characters. Has recent inquiry into the conditioned response any pertinent bearing on this vexed question ?

The exact meaning to be attached to the word "acquired" needs careful definition. The emphasis falls on some specific modification of structure impressed in some specific way on the organism in the course of its individual life. But it is often very difficult clearly to distinguish these specific modifications and the specific way in which they are induced. In our present context, however, it seems that there is less difficulty. It seems that the secondary or connecting routes in the central nervous system —those which characterise the conditioned response—are acquired in a pretty definite sense, whereas the primary routes, thus connected, imply hereditary permeability and are not in the same sense acquired. In other words, primary responses observable on the first occasion are inherited as such ; but secondary responses are the outcome of acquired permeability. The question at issue, therefore, *in this context* is this : Is there evidence that secondary permeability acquired in one generation is inherited as primary permeability in the next generation ?

Professor Pavlov has recently published some very interesting and important results, the outcome of his observations on mice. In these mice, as in Dr. Allen's fish, an " association " was formed between feeding and the sound of an electric bell. He dealt, however, with several generations of mice. In those with which he started some 300 " lessons " were required. But in the children of these mice 100 " lessons " sufficed, in their children only 30 " lessons " were required, and in *their* children but 5. The question therefore arises : May we, on the basis of this evidence, say that a conditioned response is inherited ? I think not.

The observations seem conclusively to show that specific secondary routes are more readily established in the successive generations. They do not *yet* show that a secondary route becomes a primary route—that apart from *any* lessons, even *one* lesson, auditory stimulation is followed by this response in behaviour. The number of occasions requisite to the establishment of this conditioned response is reduced in four generations from 300 to 5 ; but it is still a conditioned and not a primary response. In young chicks avoidance of nauseous things—such as medicated (quinine-soaked) rice-grains—requires only two or three lessons. But I have never observed any young bird that avoided at sight nauseous insects that must have been often tasted by their parents and grandparents.

Professor Pavlov's observations are of great interest and value. They open up the question how the reduction in the number of lessons is to be interpreted. But, so far, they afford no evidence that after so many generations a conditioned response is inherited as a response of the primary order.

Into the more general question of the transmission from parent to offspring of something that facilitates individual acquisition—and this seems to be increasingly probable—I cannot here enter. It must suffice to say that what we now know concerning the function of the endocrine glands throws new light on this difficult problem.

